# Effectiveness and safety of acupuncture in treating sleep disturbance in dementia patients: A PRISMA-compliant systematic review and limitations of current evidence

**DOI:** 10.1097/MD.0000000000026871

**Published:** 2021-08-13

**Authors:** Chan-Young Kwon, Boram Lee, Da-Jung Ha

**Affiliations:** a Department of Oriental Neuropsychiatry, Dong-eui University College of Korean Medicine, 52-57, Yangjeong-ro, Busanjin-gu, Busan, Republic of Korea; b Department of Clinical Korean Medicine, Graduate School, Kyung Hee University, 26, Kyungheedae-ro, Dongdaemun-gu, Seoul, Republic of Korea; c Clinical Research Coordinating Team, Korea Institute of Oriental Medicine, 1672, Yuseong-daero, Yuseong-gu, Daejeon, Republic of Korea; d Department of Internal Medicine, Dong-eui University College of Korean Medicine, 52-57, Yangjeong-ro, Busanjin-gu, Busan, Republic of Korea.

**Keywords:** acupressure, acupuncture, dementia, East Asian traditional medicine, sleep initiation and maintenance disorders, systematic review

## Abstract

**Background:**

Dementia is of increasing importance, as it is a major public health problem worldwide. Sleep disturbance is common in dementia patients and may be associated with worse cognitive symptoms or behavioral and psychological symptoms of dementia. Non-pharmacological approaches, such as acupuncture, for treating this clinical condition are gaining importance. This study aimed to comprehensively search and analyze randomized controlled clinical trials (RCTs) of acupuncture in treating sleep disturbance or sleep disorders in dementia patients

**Methods:**

A comprehensive search was conducted from 12 electronic databases on December 2, 2020. We included RCTs reporting the effectiveness and safety of acupuncture in treating sleep disorders or disturbance in dementia patients. The methodological quality of the included studies was assessed using the Cochrane Collaboration's risk-of-bias tool.

**Results:**

Five articles with four original RCTs met the inclusion criteria. These studies reported clinical data suggesting that adjuvant acupuncture for hypnotics, and ear acupressure in dementia patients with sleep disorders or sleep disturbance may have clinical benefits in certain sleep-related parameters and total effective rate (TER). Only 1 study reported the safety profile of the intervention, and no acupuncture-related adverse reactions were reported. Some studies compared 2 kinds of acupuncture methods, and found that specific acupuncture methods were superior to conventional acupuncture in improving sleep-related parameters, cognitive function and TER. The methodological quality of the included clinical studies was not high.

**Conclusions:**

There were limited acupuncture studies on this topic. Given the number of studies included and their sample size, methodological quality, and heterogeneities, clinically relevant conclusions could not be drawn. Further clinical studies are needed in this field considering its urgency and importance.

## 1 Introduction

Dementia is of increasing importance, as it is a major public health problem worldwide. According to the World Health Organization's predictions, it is estimated that 131.5 million people globally will suffer from dementia by 2050, and its economic cost will reach 2 trillion dollars by 2030.^[1]^ One of the important factors that increases the socioeconomic costs associated with dementia is the caregiver burden.^[2,3]^ Caregiver burden increases when family or professional caregivers face difficulties in managing dementia patients. The difficulty in managing dementia patients is often due to non-cognitive symptoms, also called behavioral and psychological symptoms of dementia (BPSD), such as depression, anxiety, aggression, anxiety, delusions, and wandering rather than cognitive symptoms, which are the core symptoms of dementia.^[4,5]^ Moreover, sleep problems in dementia patients are not only related to worsened BPSD, but also predict a poor prognosis in dementia patients^[6]^ and increase the caregiver burden.^[7]^ In general, the prevalence of insomnia increases with age, and poor sleep is associated with a high risk of cognitive decline.^[8]^ Moreover, studies have shown that the prevalence of sleep disturbance in dementia patients is about 70%, suggesting the need for effective coping strategies.^[9]^

In general, non-pharmacological strategies such as cognitive behavioral therapy for insomnia (CBT-I) are recommended for the treatment of insomnia, especially chronic insomnia, and short-term antidepressants, benzodiazepines, or Z-drugs are prescribed as needed.^[10]^ However, treatment of insomnia using these psychotropic drugs is not encouraged in the elderly.^[11]^ In particular, benzodiazepines and Z-drugs may be directly related to poor cognitive function or may cause fatal outcomes in elderly patients with dementia by increasing the risk of falls.^[12–14]^ Therefore, non-pharmacological interventions are an important strategy for the treatment of insomnia in this population.^[15–17]^ However, individuals with severely limited cognitive function may not fully benefit from CBT-I, as the treatment requires cognitive engagement. Therefore, the importance of a simple, safe, and accessible treatment strategy is emphasized in the management of insomnia in patients with dementia.

Acupuncture is a non-pharmacological intervention with origins in East Asian traditional medicine, and its safety has been generally established.^[18]^ Moreover, acupuncture is recognized as a promising therapeutic approach for insomnia.^[19]^ It also has therapeutic potential for certain conditions, including chronic pain^[20]^ and depression,^[21]^ which present a high comorbidity in elderly patients. One of the greatest benefits of conducting acupuncture treatment for insomnia in patients with dementia could be that it does not require cognitive engagement. Furthermore, these treatments are free from drug interaction problems with anti-dementia drugs, thus reducing the risk of polypharmacy in this vulnerable population.

However, clinical evidence of acupuncture for the treatment of sleep disturbance in patients with dementia has not been systematically analyzed. Therefore, in this systematic review, we comprehensively searched and analyzed randomized controlled clinical trials (RCTs) of acupuncture in treating sleep disturbance or sleep disorders in dementia patients and discussed the clinical implications and suggestions for further studies.

## 2 Methods

This review was reported in accordance with the Preferred Reporting Items for Systematic Reviews and Meta-Analyses for Acupuncture Statement^[22]^ (Supplementary file S1). Since this study is a systematic review of original articles that have already been published, ethical approval was not necessary.

### 2.1 Data sources and search strategy

One researcher (CY Kwon) searched the following electronic bibliographic databases from their inception dates to December 2, 2020: 6 English databases (MEDLINE via PubMed, EMBASE via Elsevier, the Cochrane Central Register of Controlled Trials, Allied and Complementary Medicine Database via EBSCO, Cumulative Index to Nursing and Allied Health Literature via EBSCO, and PsycARTICLES via ProQuest), 3 Korean databases (Oriental Medicine Advanced Searching Integrated System, Research Information Service System, and Korea Citation Index), and 3 Chinese databases (China National Knowledge Infrastructure, Wanfang Data, and VIP). Additionally, we searched the reference lists of the relevant articles and manually searched Google Scholar to identify additional gray literature for inclusion. Supplementary file S2 shows the search strategies for each database.

### 2.2 Inclusion criteria

#### 2.2.1 Types of studies

Original RCTs using acupuncture on sleep disturbance in dementia patients were included. There was no restriction on the publication language.

#### 2.2.2 Types of participants

Studies involving people affected with any type of dementia were included. The diagnosis of dementia was conducted clinically as well as by using validated diagnostic criteria, such as the Diagnostic and Statistical Manual of Mental Disorders, International Classification of Diseases, National Institute of Neurological and Communicative Disorders and Stroke, and Alzheimer's disease and Related Disorders Association, were allowed. There were no restrictions on the sex, age, ethnicity, or race of the participants. However, as somnolence is outside the scope of this review, studies involving patients with dementia with somnolence were excluded.

#### 2.2.3 Types of interventions

Any type of acupuncture (manual acupuncture, electroacupuncture, auriculotherapy, acupressure, etc.) performed as monotherapy or adjunctive therapy to conventional medicine, including hypnotics or usual care, was included. For control intervention, studies involving wait-list, placebo (sham-acupuncture or sham-acupressure), or other active controls including hypnotics were included. Also, studies comparing 2 or more different acupuncture methods were also allowed.

#### 2.2.4 Types of outcome measures

The primary outcomes were the Pittsburgh Sleep Quality Index (PSQI),^[23]^ a validated measure of subjective sleep quality, and polysomnography and actigraphy, which are objective measures of sleep parameters. The secondary outcomes included assessment of the cognitive symptoms (mini mental state examination [MMSE] and Alzheimer's disease assessment scale–cognitive subscale), and total effective rate (TER). Additionally, measures of activities of daily living, instrumental activities of daily living, and quality of life of dementia patients were considered as secondary outcomes. Studies in which sleep-related parameters were not reported were excluded.

### 2.3 Study selection

Two researchers (CY Kwon and B Lee) independently screened the searched articles to identify titles and/or abstracts of studies that potentially met the inclusion criteria. Following this, they independently assessed full texts of potentially eligible studies for final inclusion. Any disagreements between the researchers were resolved through discussion with other researchers (DJ Ha). EndNote X8 (Clarivate Analytics, Philadelphia, USA) was used to manage quotations of the included articles.

### 2.4 Data extraction

Two researchers (CY Kwon and B Lee) independently performed the data extraction process. A pre-defined and standardized extraction form was used to extract data from the included studies. The extracted data included the first author's name, publication year, publication country, sample size and withdrawals, details of participants, treatment and control interventions considering the template for intervention description and replication checklist,^[24]^ outcome measures, adverse events, information for assessment of the risk of bias (RoB), and funding sources. Any disagreements between the researchers were resolved through discussion with other researchers (DJ Ha). Excel 2016 (Microsoft, Redmond, WA, USA) and Dropbox (Dropbox, Inc., California, USA) folders were used to extract and share data.

### 2.5 Quality assessment

Two researchers (CY Kwon and B Lee) independently performed the quality assessment process. For included RCTs, the methodological quality was assessed using the Cochrane Collaboration's RoB tool. Seven domains of each RCT (for e.g., random sequence generation, allocation concealment, blinding of participants, personnel, and outcome assessors, completeness of outcome data, selective reporting, and other biases) were assessed as “low risk,” “unclear risk,” or “high risk”.^[25]^ In case of other bias, the statistical baseline imbalances of demographic information or clinical severity between the treatment and control groups were considered. Any disagreements between the researchers were resolved through discussion with other researchers (DJ Ha). Each evaluation was recorded in an Excel 2016 (Microsoft, Redmond, WA, USA) file and was shared using Dropbox (Dropbox, Inc., California, USA) folders. The RoB graph and summary were produced RevMan 5.4 (The Cochrane Collaboration, London, England).

### 2.6 Data analysis

Narrative synthesis of the findings from all included studies, including the demographic characteristics of the participants, details of the interventions, outcomes, and results, was performed. Due to the heterogeneity of the design of the studies involved and the interventions used, quantitative synthesis (i.e., meta-analysis) could not be performed. Since quantitative synthesis was not performed, subgroup analysis and sensitivity analysis were not possible. In addition, publication bias using a funnel plot could not be evaluated.

## 3 Results

### 3.1 Study selection

**Figure 1:**
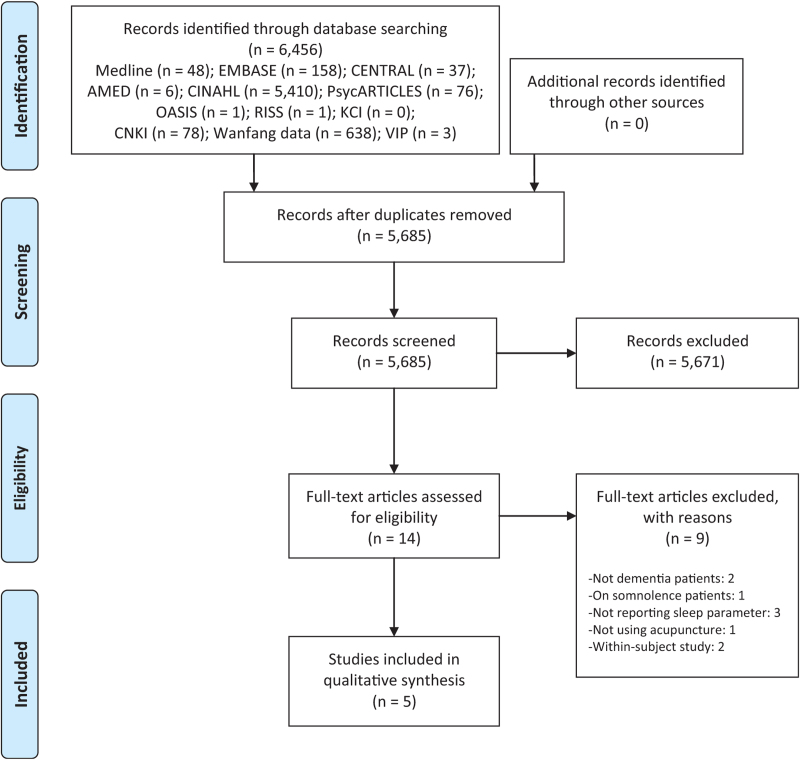
A PRISMA flow diagram of the literature screening and selection processes. AMED = Allied and Complementary Medicine Database, CENTRAL = Cochrane Central Register of Controlled Trials, CINAHL = Cumulative Index to Nursing and Allied Health Literature, CNKI = China National Knowledge Infrastructure, KCI = Korea Citation Index, OASIS = Oriental Medicine Advanced Searching Integrated System, ZSAaRISS = Research Information Service System.

Among the 5685 documents included after excluding duplications, 14 papers were selected through abstract and title screening. After further review of the full text of these papers, the following were excluded: 2 articles not on dementia patients, 1 on somnolence, 3 not reporting sleep parameters, 1 not using acupuncture, and 2 within-subject studies. Finally, 5 articles were included in this systematic review^[26–30]^ (Fig. 1).

### 3.2 Study characteristics

Of 5 articles,^[26–30]^ 2 were dissertations,^[27,28]^ and 1 dissertation^[28]^ and 1 journal article^[26]^ reported the same data. With the exception of 1 study from Spain,^[30]^ all others were conducted in China. With the exception of 1 RCT including patients with any type of dementia only,^[30]^ all others enrolled patients with both dementia and sleep disorders or sleep disturbance.^[26–29]^ All the studies included patients aged 65 years or older. Only 1 study reporting mild-to-moderate dementia^[27]^ specified the severity of dementia using the clinical dementia rating.^[27]^ Regarding the comparisons conducted, 2 RCTs (in 3 articles)^[26–28]^ compared different acupuncture strategies, including *Anshen* (pronunciation of Chinese character which means to calm the mind) acupuncture, ear acupuncture, and conventional acupuncture. One RCT compared electroacupuncture combined with hypnotics and hypnotics alone,^[29]^ while the other was a three-arm study comparing ear acupressure, relaxing massage, and usual care.^[30]^ The reported duration of treatment varied from 3 weeks to 12 weeks. Regarding the reported outcomes, the primary outcome of PSQI was used in 3 studies (in 4 articles)^[26–29]^ (Table 1).

**Table 1:** Characteristics of included studies.

Study	Sample size (included →analyzed)	Mean age (range) (yr)	Sex (male: female)	Population	Treatment intervention	Control intervention	Outcome
Li 2017Li 2019	40 (20:20)→40 (20:20)	TG: 82.8 ± 4.775 CG: 80.5 ± 4.807	TG (11:9) CG (8:12)	Alzheimer's disease (Guiding Principles for Clinical Study of New Chinese Medicines) Sleep disorder (Development of Diagnostic Criteria for Defining Sleep Disturbance in Alzheimer's Disease)	*Anshen* acupuncture	Conventional acupuncture	1. PSQI 2. TER
Lu 2020	72 (36:36)→66 (32:34)	TG: 68.79 ± 3.20 CG: 68.26 ± 3.50	TG (15:17) CG (17:17)	Alzheimer's disease (Clinical Diagnosis and Management of Alzheimer's Disease) Sleep disorder (Development of Diagnostic Criteria for Defining Sleep Disturbance in Alzheimer's Disease) ∗CDR 1–2	Ear acupuncture+conventional acupuncture	Conventional acupuncture	1. PSQI 2. MMSE 3. ESS 4. TER
Zhang 2017	82 (41:41)→82 (41:41)	TG: 66.12 ± 11.33 (61–82) CG: 65.25 ± 10.62 (60–81)	TG (23:18) CG (22:19)	Alzheimer's disease (Diagnostic and Statistical Manual of Mental Disorders) Sleep disorder (International Classification of Sleep Disorders)	Electroacupuncture+medication	Medication	1. TER 2. PSQI global score
Rodríguez-Mansilla 2013	120 (40:40:40)→111 (40:35:36)	TG: 85.4 ± 5.9 CG1: 85.8 ± 4.9 CG2: 81.9 ± 5.9	NR	Dementia (Diagnostic and Statistical Manual of Mental Disorders, 4th edition)	Ear acupressure	CG1: Relaxing massage +routine careCG2: Routine care	1. Structured questionnaire

### 3.3 Risk of bias within studies

**Figure 2:**
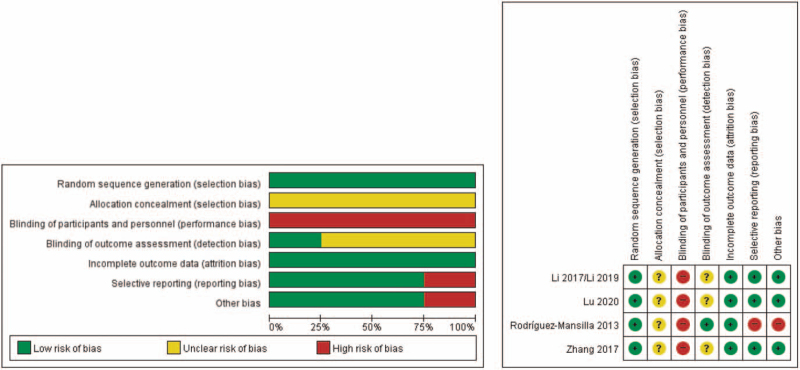
Risk of bias within studies. (A) Risk of bias graph, (B) Risk of bias summary.

All 4 RCTs (in 5 articles)^[26–30]^ reported random sequence generation using a random number table and were evaluated as having a low RoB in this item. No studies reported the allocation concealment. Three RCTs (in 4 articles)^[26–29]^ did not report the blinding of the participants, personnel, or outcome assessors; the other RCT^[30]^ reported that the participants and outcome assessors were blinded, but not the personnel. Two RCTs (in 3 articles)^[26,28,29]^ reported no dropout of participants, but the other 2 RCTs^[27,30]^ reported dropouts. However, the number was similar between the groups, and the dropping-out did not appear to be correlated with the intervention. Three RCTs (in 4 articles)^[26–29]^ reporting PSQI scores were rated as low RoB in the selective reporting item, and the other RCT^[30]^ using only a self-produced questionnaire was rated as having a high RoB. In 3 RCTs (in 4 articles),^[26–29]^ baseline statistical similarity was reported and evaluated as low risk in the other sources of bias items. However, the other RCT^[30]^ was evaluated as high risk in this item because a statistically significant difference was reported in the participants’ ages between the groups at baseline, and there was no mention of statistical considerations such as using participant age as a covariate when analyzing the results (Fig. 2).

### 3.4 Effectiveness and safety of acupuncture

#### 3.4.1 Effectiveness

In the study by Zhang (2017),^[29]^ 82 Alzheimer's disease (AD) patients with sleep disorders were randomized into 2 groups treated with electroacupuncture combined with 7.5 mg per day of midazolam (combined treatment group) and midazolam alone (control group), respectively, for 30 days (treatment frequency: daily). The PSQI and TER results were reported. The combined treatment group showed significantly better improvement in the PSQI global score (*P* < .05) as compared to the control group. Furthermore, the TER results of the combined treatment group (37/41, 90.24%) were significantly higher than that of the control group (24/41, 58.54%) (*P* < .05). In the study by Rodríguez-Mansilla (2013),^[30]^ 120 dementia patients receiving routine care were randomized into 3 groups treated with ear acupressure, relaxing massage, and routine care alone, respectively, for 12 weeks (treatment frequency: daily self-acupressure for ear acupressure). The results of the structured questionnaire were reported. As results, acupressure groups showed significant differences in the percentage of participants complaining of behavioral alterations (*P* = .000), sleep disturbance (*P* = .000), participation in therapy (*P* = .000), and participation in eating (*P* = .000) when compared with the routine care group (Table 2).

**Table 2:** Details of interventions used and results.

Study	Treatment intervention	Control intervention	Treatment duration	Results reported	Side effect
Li 2017Li 2019	1. Anshen acupunctureGV20, GV11, GV10, GV9, (bilaterally) BL15, HT7, BL18, BL20, BL23Needle retention: 30 minOne treatment course: 6 consecutive days + 1 d off	1. Conventional acupunctureEX-HN1, (bilaterally) SP6, HT7, Anmian (extra), KI6, BL62, KI3, GB39, ST36Needle retention: 30 minOne treatment course: 6 consecutive days + 1 d off	3 wks (total 18 sessions)	1. PSQI 1) sleep quality: TG (0.550 ± 0.826) < CG (1.500 ± 1.000), *P* = .002 2) sleep onset latency: TG (0.550 ± 0.759) <CG (1.700 ± 0.865), *P* = .001 3) sleep duration: TG (0.800 ± 1.005)<CG(1.450 ± 0.945), *P* = .042 4) sleep efficiency: TG(1.150 ± 0.875)<CG (1.650 ± 1.040), *P* = .108 5) sleep disturbance: TG (0.700 ± 0.923)<CG (1.400 ± 0.883), p = 0.019 6) use of hypnotic: TG(0.850 ± 0.813)<CG(1.500 ± 1.147), p = 0.046 7) daytime dysfunction: TG(0.950 ± 0.999)<CG(1.300 ± 1.031), p = 0.282 8) global score: TG(5.600 ± 4.650)<CG(10.500 ± 5.226), *P* = .0032. TER: TG(18/20)>CG(15/20), *P* < .05	TG: none CG: none
Lu 2020	1. Ear acupuncture(unilaterally) Shenmen, subcortical, Anmian, heart, kidneyPress needle attachedDaily self-acupressure: 1 min/3 timesLeft and right shift once every two days2. Conventional acupuncture	1. Conventional acupunctureGV20, GV16, GV14, GV4, GV3, (bilaterally) Anmian(extra), SP6, KI3, ST36Needle retention: 30 minOne treatment course: 6 consecutive days + 1 d off	4 wks (total 24 sessions)	1. PSQI 1) sleep quality: TG (0.97 ± 0.49) < CG (1.38 ± 0.77), *P* < .05 2) sleep onset latency: TG(0.78 ± 0.59)<CG (1.32 ± 0.85), *P* < .01 3) sleep duration: TG(1.43 ± 0.67)<CG(1.44 ± 0.57), p < 0.05 4) sleep efficiency: TG(0.91 ± 0.74)<CG (1.38 ± 0.50), *P* < .01 5) sleep disturbance: TG (0.94 ± 0.56) < CG (1.38 ± 0.57), *P* < .01 6) daytime dysfunction: TG (0.84 ± 0.68)<CG (1.47 ± 0.68), *P* < .01 8) global score: TG (5.47 ± 2.19)<CG (13.94 ± 2.05), *P* < .012. MMSE: TG (21.22 ± 2.31)>CG (19.88 ± 1.54), *P* = .0053. ESS: TG (4.41 ± 1.41) <CG (6.24 ± 1.32), *P* = .0004. TER: TG (28/32)>CG (26/34), *P* < .05	NR
Zhang 2017	1. ElectroacupunctureGV20, GV24, EX-HN1, (bilaterally) Anmian(extra), Taiyang(extra), P6, HT7, SP6, KI1EA: Taiyang-EX-HN1, 2–100 Hz, 2–4 VNeedle retention: 25 minOne treatment course: 10 consecutive days2. Medication	1. MedicationMidazolam maleate 7.5 mg 1T hs	30 d (total 30 sessions)	1. TER: TG (37/41)>CG (24/41), *P* < .05 2. Global score of PSQI: TG (1.59 ± 2.15)<CG(4.15 ± 1.77), *P* < .05	NR
Rodríguez-Mansilla 2013	1. Ear acupressureShenmen, 159 Muscle relaxant (located in peripheral inferior concha), heartVaccariae Semen attachedDaily self-acupressureReplace every 15 d2. Routine care	CG1: 1. Relaxing massageOne treatment course: 5 consecutive days + 2 days offTreatment time: 20 min2. Routine careCG2: 1. Routine care	12 wks	1. Structured questionnaire 1) Behavior alterations: TG(3/40)>CG1 (0/35), *P* = .098The difference between the three groups, *P* = .000; CG2(34/36) 2) Sleep disturbances: TG (3/40)>CG1 (1/35), *P* = .372The difference between the three groups, *P* = .000; CG2 (36/36) 3) Participation in therapy: TG(34/40)<CG1(34/35), *P* = .314The difference between the three groups, *P* = .000; CG2(5/36) 4) Participation in eating: TG (40/40)>CG1(33/35), *P* = .568The difference between the three groups, *P* = .000; CG2 (18/36)	NR

#### 3.4.2 Safety

Only 1 study (in 2 articles) reported the safety profile of acupuncture.^[26,28]^ There was no report of adverse events caused by acupuncture in the study (Table 2).

### 3.5 Comparison of 2 acupuncture interventions

In the study by Li (2017),^[26,28]^ 40 AD patients with sleep disorders were randomized into 2 groups treated with *Anshen* acupuncture and conventional acupuncture (control group), respectively, for 3 weeks (treatment frequency: 6 times per week). The PSQI and TER results were reported. As compared to the control group, the *Anshen* acupuncture group showed significantly better improvement in the PSQI global score (*P* = .003) and in some sub-items including sleep quality (*P* = .002), sleep onset latency (*P* = .001), sleep duration (*P* = .042), sleep disturbance (*P* = .019), and use of hypnotics (*P* = .046). There were no significant differences between the groups in sleep efficiency (*P* = .108) and daytime dysfunction (*P* = .282). Moreover, the *Anshen* acupuncture group (18/20, 90.0%) showed significantly higher TER as compared to the control group (15/20, 75.0%) (*P* < .05). In the study by Lu (2020),^[27]^ 72 mild-to-moderate (CDR, 1–2) AD patients with sleep disorders were randomized into 2 groups treated with ear acupuncture combined with conventional acupuncture (combined acupuncture group) and conventional acupuncture alone (control group), respectively, for 4 weeks (treatment frequency: daily self-acupressure for ear acupuncture; and 6 times per week for conventional acupuncture). As an emergency medication, 3.75 mg per day of zopiclone was administered to all patients. The PSQI, MMSE, Epworth Sleepiness Scale (ESS), and TER scores were reported. As compared to the control group, the combined acupuncture group showed significantly better improvement in the PSQI global score (*P* < .01) and in all sub-items, including sleep quality (*P* < .05), sleep onset latency (*P* < .01), sleep duration (*P* < .05), sleep efficiency (*P* < .01), sleep disturbance (*P* < .01), and daytime dysfunction (*P* < .01). Moreover, the results of MMSE were significantly higher (*P* = .005) and that of ESS were significantly lower (*P* = .000) in the combined acupuncture group as compared to the control group. The combined acupuncture group (28/32, 87.5%) showed significantly higher TER results as compared to the control group (26/34, 76.47) (*P* < .05) (Table 2).

## 4 Discussion

### 4.1 Summary of evidence

The studies included in this review have reported clinical data suggesting that adjuvant acupuncture for hypnotics, and ear acupressure in dementia patients with sleep disorders or sleep disturbance, may have clinical benefits on some sleep-related parameters (i.e., PSQI and structured questionnaire) and TER. Only 1 study reported the safety profile of the intervention. Some studies compared 2 kinds of acupuncture methods, and found that specific acupuncture methods (i.e., *Anshen* acupuncture and ear acupuncture combined with conventional acupuncture) were superior to conventional acupuncture in improving some sleep-related parameters (i.e., PSQI and ESS), MMSE and TER. However, in the evaluation of the methodological quality, using the Cochrane RoB tool showed that none of the RCTs presented all 7 items with low RoB. All included RCTs^[26–30]^ seemed to have used an appropriate randomization method, but lacked allocation concealment and blinding procedures. These methodological limitations are important because they increase the likelihood of overestimating the effectiveness of the interventions used. Given the small number of studies included and their small sample size and low methodological quality, this review could not draw definite conclusions about the effectiveness and safety of acupuncture or superiority of certain acupuncture treatment methods in dementia patients with sleep disturbance.

### 4.2 Strengths and limitations

This review explores the effectiveness and safety of acupuncture, a potentially promising non-pharmacological treatment strategy, in sleep disturbance, which is 1 of the important clinical challenges in dementia patients. However, the role of acupuncture has rarely been discussed in previous articles that have emphasized the importance of non-pharmacological treatments for this condition.^[15–17]^ Since complementary and alternative medicine, including acupuncture, is already widely used in geriatric care, and the need for an evidence base to demonstrate their efficacy and safety has been pointed out,^[31]^ this systematic review sought to gather comprehensive clinical evidence for these interventions.

However, the following limitations should be considered when interpreting the results of this review. **First**, one of the biggest limitations of this review was the small number of studies included. This means that research in this field is highly limited. In addition, among the included RCTs, only 1 study compared hypnotics and acupuncture,^[29]^ and the clinical relevance of existing RCTs in this field was generally low. No clinical study compared the effectiveness of acupuncture and CBT-I, which is initially recommended for the treatment of insomnia in the elderly.^[10]^ Therefore, the clinical studies included in this review may have limited influence on the decision-making of clinicians, patients, and/or caregivers from the perspective of evidence-based medicine (EBM). **Second**, the methodological quality of the included studies was not very high. In particular, the lack of allocation concealment and blinding of participants and personnel or outcome assessors were likely to contribute to an overestimation of effect size. The overall small sample size of the included studies could also contribute to the overestimation. **Third**, the included studies rarely considered the type of dementia. Studies investigating certain types of dementia focused primarily on patients with AD.^[26–29]^ However, existing evidence has reported that sleep disturbance may differ between different types of dementia.^[32–34]^ These may reflect differences in the underlying biological mechanisms associated with sleep disturbance in patients with dementia, potentially affecting treatment outcomes. In addition, there was only 1 study specifying the severity of dementia in the included studies.^[27]^ To the best of our knowledge, only few studies have investigated the differences in sleep disturbance according to dementia severity, but differences in psychiatric symptoms as well as cognitive function do exist,^[35]^ which can potentially affect treatment outcomes. **Fourth**, the included clinical studies lacked the use of consistent outcomes. Furthermore, the lack of an optimal assessment method for evaluating sleep disturbance in patients with dementia is a major task in this field of research. Considering the difficulty in applying the self-reporting scale in patients with dementia, especially those with moderate to severe dementia, a scale evaluated by the caregiver can be considered. This approach is important, because caregiver burden is another important issue related to dementia.^[7]^ Actigraphy may still be a promising strategy for assessing sleep in this population. For example, although it is a within-subject study that is not included in this review, Kwok (2013) using ear acupressure evaluated sleep parameter using wrist actigraphy in mild AD patients with insomnia.^[36]^**Fifth**, standardization of the interventions used was insufficient. Especially, the acupoints (i.e., treatment sites) used for acupuncture were also inconsistent between the studies. This heterogeneity emphasizes that an ideal treatment strategy for acupuncture has not been established in terms of treatment dose and frequency to exert an optimal effect.

### 4.3 Suggestions for further studies

It is important to establish the evidence base for non-pharmacological intervention in terms of EBM to improve the management of dementia and alleviate the associated burden. We would like to present the following suggestions for future research in this field based on the major limitations of our review. **First**, the number of acupuncture studies on sleep disorders or sleep disturbance in patients with dementia was insufficient. However, the effectiveness of acupuncture on the core symptoms of dementia has been reported sufficiently.^[37,38]^ Considering that application of complementary and alternative medicine is common in elderly patients,^[31]^ further studies on acupuncture for sleep problems or sleep disturbance in patients with dementia need to be conducted. It cannot be denied that the highest level of evidence has been obtained from RCTs; however, as of the current state of evidence, case series or within-subject studies that have been well reported are also needed to establish the evidence base. These studies should provide either a complementary/alternative role of acupuncture for CBT-I or medications that are currently being used to treat sleep problems and sleep disturbance in elderly or dementia patients, if possible. **Second**, clinical studies with stringent methodological quality should be conducted in this field. Ideally, clinical studies with sufficient high power should be conducted by calculation of the sample size. Small-scale studies of poor methodological quality may overestimate the effect size of acupuncture, resulting in unreliable evidence. **Third**, differences between the various types of dementia should be considered during the recruitment or analysis phases of the study. Moreover, the severity of dementia is an important parameter that cannot be overlooked. There are many other factors to consider when analyzing the effects of acupuncture on sleep problems in dementia patients. Since insomnia in dementia patients may be related to clinical aspects of dementia such as cognitive symptoms and BPSD,^[6]^ both direct effects of acupuncture for those outcomes and indirect effects mediated by sleep parameters should be considered in the analysis. Conversely, acupuncture may improve the sleep status in dementia patients by mediating factors that interfere with sleep quality, such as chronic pain. However, these hypotheses should be verified through further clinical studies. **Fourth**, since the self-reporting scale for sleep disturbance depends on the patient's cognitive function and memory, it is ideal to obtain information from both patients with dementia and the caregiver. Objective evaluation measures, including actigraphy and polysomnography, occupy an important position in the field of sleep research, and they can be used for dementia patients. In addition to subjective and objective sleep-related parameters, the evaluation of related caregiver burden and cost-effectiveness should be considered. **Fifth**, in the acupuncture research field, one of the main causes of heterogeneity between studies is the lack of intervention. Thus, establishing an optimal acupuncture strategy, at least at the basic level, guarantees to address these non-pharmacological interventions mainly from the EBM perspective. It may be promising to establish a standardized acupuncture strategy around acupoints that are biologically supported at the clinical or preclinical levels. For example, a recent study investigated the effect of acupuncture on ST36 and GV20 in rats with vascular dementia and analyzed the contribution of various therapeutic parameters, including manipulation, retention, and frequency of acupuncture.^[39]^ It was found that the Morris water maze results and hippocampal neuronal integrity in the needle retention for 10 minutes, rotation for 30 second every 5 minutes, or daily treatment acupuncture groups were better than those in the non-retention, non-rotation, or alternative day treatment groups.^[39]^

## 5 Conclusion

This systematic review comprehensively searched and analyzed RCTs of acupuncture in treating sleep disturbance or sleep disorders in dementia patients. Some studies have reported clinical data suggesting that adjuvant acupuncture for hypnotics, and ear acupressure in the population may have clinical benefits on some sleep-related parameters. Also, some other studies compared 2 kinds of acupuncture methods, and found that specific acupuncture methods were superior to conventional acupuncture in improving sleep-related parameters, cognitive function and TER. However, given the number of studies included and their sample size, methodological quality, and heterogeneities, this review cannot draw any clinically relevant conclusions. Considering the urgency and importance of this area, additional clinical studies should be conducted, taking into account the limitations discussed in this review.

## Author contributions

**Conceptualization:** Chan-Young Kwon.

**Funding acquisition:** Chan-Young Kwon.

**Methodology:** Chan-Young Kwon, Boram Lee, Da-Jung Ha.

**Supervision:** Chan-Young Kwon.

**Writing – original draft:** Chan-Young Kwon, Boram Lee.

**Writing – review & editing:** Chan-Young Kwon.

## Supplementary Material

Supplemental Digital Content

## Supplementary Material

Supplemental Digital Content
