# Supplementary material for: Effectiveness and safety of acupuncture in treating sleep disturbance in dementia patients: A PRISMA-compliant systematic review and limitations of current evidence
Source: Medicine (Baltimore). 2021 Aug 13;100(32):e26871. doi: 10.1097/MD.0000000000026871 (PMC8360405; doi:10.1097/MD.0000000000026871)
Supplement: Supplemental Digital Content [file medi-100-e26871-s002.docx]

**Supplemental digital content 2. Search strategies used in each database**

**Medline via PubMed**

|  | Searches | Results |
| --- | --- | --- |
| #1 | Dementia[MeSH] OR dement* OR Alzheimer* OR “Lewy body” OR Huntington* OR Parkinson* OR “Pick disease” OR “cognitive impairment” | 433,359 |
| #2 | Sleep[MeSH] OR sleep wake disorders[MeSH] OR sleep* OR insomnia* OR wakeful* OR sleepless* OR dyssomn* | 260,019 |
| #3 | Acupuncture[MeSH] OR Acupressure[MeSH[ OR “Acupuncture Therapy”[MeSH] OR “Acupuncture Points”[MeSH] OR acupunct* OR electroacupunct* or electro-acupunct* OR acupoint* OR acupressure OR auriculotherapy | 35,619 |
| #4 | #1 AND #2 AND 3 | **48** |

**EMBASE via Elsevier**

|  | Searches | Results |
| --- | --- | --- |
| #1 | 'dementia'/exp OR ‘dement*’ OR ‘Alzheimer*’ OR ‘Lewy body’ OR ‘Huntington*’ OR ‘Parkinson’ OR ‘Pick disease’ OR ‘cognitive impairment’ | 703,599 |
| #2 | ‘sleep’/exp OR ‘sleep’ OR ‘sleep disorder’/exp OR ‘sleep disorder’ OR ‘insomnia*’ OR ‘wakeful*’ OR ‘sleepless*’ OR ‘dyssomn*’ | 473,007 |
| #3 | ‘acupucnutre’/exp OR ‘acupunct*’ OR ‘electroacupunct*’ OR ‘electro-acupunct*’ OR ‘acupoint*’ OR ‘acupressure’ OR ‘auriculotherapy’ | 55,814 |
| #4 | #1 AND #2 AND #3 | **158** |

**CENTRAL**

|  | Searches | Results |
| --- | --- | --- |
| #1 | MeSH descriptor: [Dementia] explode all trees | 5,936 |
| #2 | dement* OR Alzheimer* OR Lewy body OR Huntington* OR Parkinson* OR Pick disease OR cognitive impairment | 45,097 |
| #3 | MeSH descriptor: [Sleep] explode all trees | 5,671 |
| #4 | MeSH descriptor: [Sleep Wake Disorders] explode all trees | 8,157 |
| #5 | sleep* OR insomnia* OR wakeful* OR sleepless* OR dyssomn* | 47,885 |
| #6 | MeSH descriptor: [Acupuncture] explode all trees | 151 |
| #7 | MeSH descriptor: [Acupressure] explode all trees | 352 |
| #8 | acupunct* OR electroacupunct* or electro-acupunct* OR acupoint* OR acupressure OR auriculotherapy | 19,252 |
| #9 | (#1 OR #2) AND (#3 OR #4 OR #5) AND (#6 OR #7 OR #8) in Trials | **37** |

**AMED via EBSCO**

|  | Searches | Results |
| --- | --- | --- |
| #1 | SU Dementia OR TX dement* OR TX Alzheimer* OR TX Lewy body OR TX Huntington* OR TX Parkinson OR TX Pick disease OR TX cognitive impairment | 6,915 |
| #2 | SU Sleep OR SU Sleep wake disorders OR TX sleep* OR TX insomnia* OR TX wakeful* OR TX sleepless* OR TX dyssomn* | 3,557 |
| #3 | SU Acupuncture OR SU Acupressure OR TX acupunct* OR TX electroacupunct* OR TX electro-acupunct* OR TX acupoint* OR TX acupressure OR TX auriculotherapy | 11,895 |
| #4 | #1 AND #2 AND #3 | **6** |

**CINAHL via EBSCO**

|  | Searches | Results |
| --- | --- | --- |
| #1 | SU Dementia OR TX dement* OR TX Alzheimer* OR TX Lewy body OR TX Huntington* OR TX Parkinson OR TX Pick disease OR TX cognitive impairment | 257,336 |
| #2 | SU Sleep OR SU Sleep wake disorders OR TX sleep* OR TX insomnia* OR TX wakeful* OR TX sleepless* OR TX dyssomn* | 199,566 |
| #3 | SU Acupuncture OR SU Acupressure OR TX acupunct* OR TX electroacupunct* OR TX electro-acupunct* OR TX acupoint* OR TX acupressure OR TX auriculotherapy | 37,464 |
| #4 | #1 AND #2 AND #3 | **5,410** |

**PsycARTICLES via ProQuest**

|  | Searches | Results |
| --- | --- | --- |
| #1 | SU(Dementia) OR ‘dement*’ OR ‘Alzheimer*’ OR ‘Lewy body’ OR ‘Huntington*’ OR ‘Parkinson’ OR ‘Pick disease’ OR ‘cognitive impairment’ | 28,389 |
| #2 | SU(Sleep) OR SU(Sleep wake disorders) OR 'sleep*' OR 'insomnia*' OR 'wakeful*' OR 'sleepless*' OR 'dyssomn*' | 16,826 |
| #3 | SU(Acupuncture) OR SU(Acupressure) OR ‘acupunct*’ OR ‘electroacupunct*’ OR ‘electro-acupunct*’ OR ‘acupoint*’ OR ‘acupressure’ OR ‘auriculotherapy’ | 368 |
| #4 | #1 AND #2 AND #3 | **76** |

**OASIS**

|  | Searches | Results |
| --- | --- | --- |
| #1 | 치매 AND 불면 | 0 |
| #2 | 치매 AND 수면 | 1 |
| #3 | #1 OR #2 | **1** |

**RISS**

|  | Searches | Results |
| --- | --- | --- |
| #1 | 치매 AND 불면 AND 침 | 0 |
| #2 | 치매 AND 수면 AND 침 | 1 |
| #3 | 치매 AND 불면 AND 지압 | 0 |
| #4 | 치매 AND 수면 AND 지압 | 0 |
| #5 | #1 OR #2 OR 3 OR 4 | **1** |

**KCI**

|  | Searches | Results |
| --- | --- | --- |
| #1 | 치매 AND 불면 AND 침 | 0 |
| #2 | 치매 AND 수면 AND 침 | 0 |
| #3 | 치매 AND 불면 AND 지압 | 0 |
| #4 | 치매 AND 수면 AND 지압 | 0 |
| #5 | #1 OR #2 | **0** |

**CNKI**

|  | Searches | Results |
| --- | --- | --- |
| #1 | (SU='痴呆'+'阿尔茨海默病'+'认知障碍'+'dementia'+'Alzheimer') AND (SU='失眠'+'不寐'+'不眠'+'不睡'+'不得眠'+'不得卧'+'睡眠'+'insomnia'+'sleep disorder') AND (SU='针'+'鍼'+'acupuncture'+'指压'+'按压'+'穴位按摩'+'acupressure') | **78** |

**Wanfang data**

|  | Searches | Results |
| --- | --- | --- |
| #1 | (主题:痴呆 or 主题:阿尔茨海默病 or 主题:认知障碍 or 主题:dementia or 主题:Alzheimer) and (主题:失眠 or 主题:不寐 or 主题:不眠 or 主题:不睡 or 主题:不得眠 or 主题:不得卧 or 主题:睡眠 or 主题:insomnia or 主题:sleep disorder) and (主题:针 or 主题:鍼 or 主题:acupuncture or 主题:指压 or 主题:按压 or 主题:穴位按摩 or 主题:acupressure) | **639** |

**VIP**

|  | Searches | Results |
| --- | --- | --- |
| #1 | (M=(痴呆 OR 阿尔茨海默病 OR 认知障碍 OR dementia OR Alzheimer)) AND (M=(失眠 OR 不寐 OR 不眠 OR 不睡 OR 不得眠 OR 不得卧 OR 睡眠 OR insomnia OR sleep disorder)) AND (M=(针 OR 鍼 OR acupuncture OR 指压 OR 按压 OR 穴位按摩 OR acupressure)) | **3** |
